# FAPI-74 PET/CT Using Either ^18^F-AlF or Cold-Kit ^68^Ga Labeling: Biodistribution, Radiation Dosimetry, and Tumor Delineation in Lung Cancer Patients

**DOI:** 10.2967/jnumed.120.245084

**Published:** 2021-02

**Authors:** Frederik L. Giesel, Sebastian Adeberg, Mustafa Syed, Thomas Lindner, Luis David Jiménez-Franco, Eleni Mavriopoulou, Fabian Staudinger, Eric Tonndorf-Martini, Sebastian Regnery, Stefan Rieken, Rami El Shafie, Manuel Röhrich, Paul Flechsig, Andreas Kluge, Annette Altmann, Jürgen Debus, Uwe Haberkorn, Clemens Kratochwil

**Affiliations:** 1 Department of Nuclear Medicine, University Hospital Heidelberg, Heidelberg, Germany; 2 Heidelberg Institute of Radiation Oncology, Heidelberg, Germany; 3 Heidelberg Ion-Beam Therapy Center, Heidelberg, Germany; 4 Department of Radiation Oncology, University Hospital Heidelberg, Heidelberg, Germany; 5 Clinical Cooperation Unit Radiation Oncology, German Cancer Research Center, Heidelberg, Germany; 6 ABX-CRO Advanced Pharmaceutical Services Forschungsgesellschaft mbH, Dresden, Germany; 7 Department of Radiation Oncology, University Hospital Göttingen, Göttingen, Germany; and; 8 Clinical Cooperation Unit Nuclear Medicine, German Cancer Research Center, Heidelberg, Germany

**Keywords:** FAPI PET/CT, cancer-associated fibroblasts, lung cancer, radiation dosimetry, cold kit

## Abstract

^68^Ga-fibroblast activation protein inhibitors (FAPIs) 2, 4, and 46 have already been proposed as promising PET tracers. However, the short half-life of ^68^Ga (68 min) creates problems with manufacture and delivery. ^18^F (half-life, 110 min) labeling would result in a more practical large-scale production, and a cold-kit formulation would improve the spontaneous availability. The NOTA chelator ligand FAPI-74 can be labeled with both ^18^F-AlF and ^68^Ga. Here, we describe the in vivo evaluation of ^18^F-FAPI-74 and a proof of mechanism for ^68^Ga-FAPI-74 labeled at ambient temperature. **Methods:** In 10 patients with lung cancer, PET scans were acquired at 10 min, 1 h, and 3 h after administration of 259 ± 26 MBq of ^18^F-FAPI-74. Physiologic biodistribution and tumor uptake were semiquantitatively evaluated on the basis of SUV at each time point. Absorbed doses were evaluated using OLINDA/EXM, version 1.1, and QDOSE dosimetry software with the dose calculator IDAC-Dose, version 2.1. Identical methods were used to evaluate one examination after injection of 263 MBq of ^68^Ga-FAPI-74. **Results:** The highest contrast was achieved in primary tumors, lymph nodes, and distant metastases at 1 h after injection, with an SUV_max_ of more than 10. The effective dose per a 100-MBq administered activity of ^18^F-FAPI-74 was 1.4 ± 0.2 mSv, and for ^68^Ga-FAPI-74 it was 1.6 mSv. Thus, the radiation burden of a diagnostic ^18^F-FAPI-74 PET scan is even lower than that of PET scans with ^18^F-FDG and other ^18^F tracers; ^68^Ga-FAPI-74 is comparable to other ^68^Ga ligands. FAPI PET/CT supported target volume definition for guiding radiotherapy. **Conclusion:** The high contrast and low radiation burden of FAPI-74 PET/CT favor multiple clinical applications. Centralized large-scale production of ^18^F-FAPI-74 or decentralized cold-kit labeling of ^68^Ga-FAPI-74 allows flexible routine use.

Fibroblast activation protein (FAP) is highly expressed in the stroma of a variety of human cancers and is therefore considered promising for guiding targeted therapy (1). Quinoline-based FAP inhibitors (FAPIs) specifically bind to the enzymatic domain of FAP and are then internalized (2). Methods for conjugation of quinoline-based FAP ligands with chelators suitable for radiolabeling with various radiometals were developed (3,4). Labeled with the positron emitter ^68^Ga, these novel FAP-targeted tracers demonstrated tumor-to-nontumor contrast ratios that were equal to or even higher than those attained with ^18^F-FDG PET/CT (5).

Although ^68^Ga is available via approved ^68^Ge/^68^Ga generators, which allow batch production of approximately 2–3 patient doses per elution, the relatively short half-life of ^68^Ga (68 min, 1.90-MeV positron energy) poses some disadvantages with respect to production capacity and nuclear decay properties. The short half-life mandates in-house production, making delivery to remote centers challenging. In large centers with high patient throughput, several productions per day are required to meet potential demands, occupying a skilled workforce of radiochemists and radiopharmacists over a protracted period of the work day. If ^68^Ga-FAPI PET were to replace ^18^F-FDG PET in clinical routine, multiple generators would be needed, thus multiplying costs. Labeling with ^18^F (half-life, 110 min, 0.65-MeV positron energy) would solve these issues. Centers with an on-site cyclotron can produce ^18^F at moderate cost, and commercial sites can distribute ^18^F-labeled compounds over a wide metropolitan area, eliminating the need for on-site radiochemistry (6). The lower positron energy of ^18^F could theoretically improve spatial resolution (7).

As described in a dedicated chemistry/preclinical article (submitted for publication simultaneously), attempts to label FAPIs with covalently attached ^18^F were initially unsuccessful by demonstrating poor tumor uptake. In contrast, chelation of AlF, an approach that was proposed several years ago and has now been optimized with regard to labeling yield and specific activity (8), presented favorable results in combination with the NOTA-containing FAPI-74. The NOTA chelator also allows chelation with ^68^Ga at room temperature, which would also simplify local on-demand production in centers that already own a ^68^Ge/^68^Ga generator.

The aim of this work is to analyze the time-dependent tumor uptake and tracer biodistribution and to estimate absorbed dose for ^18^F-FAPI-74 PET/CT scans using examinations that were done under a medical indication to assist tumor volume delineation for guiding radiotherapy in lung cancer patients. In addition, we demonstrate proof of mechanism for ^68^Ga-FAPI-74 PET/CT after tracer labeling at ambient temperature.

## MATERIALS AND METHODS

### Patients

This analysis includes 10 patients (4 male, 6 female) with lung cancer (8 with adenocarcinoma, 2 with squamous cell carcinoma) and a median age of 65 y (range, 45–77 y). All patients gave written informed consent to undergo ^68^Ga-FAPI-74 PET/CT following national regulations and the Declaration of Helsinki. The radiopharmaceutical was produced in accordance with the German Pharmaceuticals Act, §13(2b). All patients were referred by a radiation oncologist, in order to improve tumor delineation for radiotherapy planning of central pulmonary lesions that would presumably have been challenging to discriminate from the myocardium with ^18^F-FDG PET. The retrospective evaluation of data acquired under clinical indication was approved by the ethical committee of Heidelberg University (permit S016/2018).

### Radiopharmaceuticals

Chelation with ^18^F-AlF was performed using the method of McBride et al. (8) as follows: 2–10 GBq of ^18^F fluoride (ZAG Cyclotron AG) in 4 mL of water were trapped on an anion exchange cartridge (Waters Accel Plus QMA Light cartridge, preconditioned with 5 mL of 0.5 M NaOAc, pH 3.9, and 10 mL of water) and eluted with 0.30 mL of 0.5 M NaOAc, pH 3.9. The solution was incubated with 6 μL of AlCl3 in water (10 mM) and 300 μL of dimethyl sulfoxide (Simga-Aldrich) for 5 min at room temperature before 20 μL of a solution of FAPI-74 (4 mM) were added. The reaction was performed at 95°C for 15 min, cooled to room temperature, diluted with 5 mL of water, and worked up by solid-phase extraction (Waters Oasis HLB Plus Light cartridge). The final product was eluted with 0.5 mL of ethanol and 5 mL of 0.9% saline and spiked with phosphate buffer before sterile filtration (Filtropur S 0.2; Sarstedt).

Chelation with ^68^Ga was achieved by adding 1.00 mL of ^68^Ge/^68^Ga generator eluate (0.6 M hydrochloric adic; ∼1.2 GBq) to a mixture of 15 μL of FAPI-74 solution (4 mM in water), 310 μL of sodium acetate (2.5 M in water), and 0.50 mL of ethanol. After incubation for 15 min at room temperature, the reaction was worked up by solid-phase extraction as described for ^18^F-FAPI-74.

### PET/CT Imaging

All imaging was performed on a Biograph mCT Flow scanner (Siemens). PET was performed in 3-dimensional mode (matrix, 200 × 200) using FlowMotion (Siemens). The emission data were corrected for randoms, scatter, and decay. Reconstruction was performed with ordered-subset expectation maximization using 2 iterations and 21 subsets, as well as Gauss filtering to a transaxial resolution of 5 mm in full width at half maximum; attenuation was corrected using the unenhanced low-dose CT images. The CT scans were reconstructed to a slice thickness of 5 mm and an increment of 3 mm using a soft-tissue reconstruction kernel (B30) with CareDose (Siemens). All patients were imaged at 10 min, 1 h, and 3 h after injection of either 259 ± 26 MBq (range, 198–290 MBq) of ^18^F-FAPI-74 (in 10 patients) or 263 MBq of ^68^Ga-FAPI-74 (in 1 patient).

### FAPI-Based Target Volume of Primary Tumors

The acquired ^18^F-FAPI-74 PET/CT examinations were used to assist tumor volume delineation for guiding radiotherapy in patients with lung cancer, similar to previous use of ^18^F-FDG PET/CT (9,10). Target volume was defined using Siemens Syngo.via software (Siemens). CT-based gross tumor volumes (GTVs) were contoured on soft-tissue and lung windows using contrast-enhanced examinations. PET-based GTVs (FAPI GTVs) were assessed by comparing tumor SUVs with healthy surrounding tissue using Syngo’s auto-contour algorithm at various SUV thresholds. Two segmentation approaches were considered: either the background level multiplied by a certain number or the percentage of SUV_max_. Contours were manually adjusted, checked for plausibility, and corrected for false-positive or -negative uptake by 2 experienced radiation oncologists and 2 nuclear medicine physicians, board-certified in their respective specialties. In clinical practice, because defining the radiation field is inherently a subjective task, a consensus of expert readers is usually considered the best applicable standard of reference.

### Biodistribution

For calculation of the SUV, circular regions of interest were drawn around the tumor lesions with focally increased uptake in transaxial slices and automatically adapted to a 3-dimensional volume of interest (VOI) with e.soft software (Siemens) at a 40% isocontour. The tracer biodistribution in patients was quantified by SUV_mean_ and SUV_max_ at 10 min, 1 h, and 3 h after injection of ^18^F-FAPI-74. The normal organs (brain, oral mucosa, parotid, thyroid, lung, heart muscle, aortic lumen content, liver, pancreas, spleen, kidney, colon, muscle, fat, and spinal cord) were evaluated with a 2-cm sphere placed inside the organ parenchyma. Statistical analysis and graphic output were performed with SigmaPlot.

### Radiation Dosimetry Estimate

The dosimetry analysis was performed using the QDOSE dosimetry software suite, version 1.1.4 (ABX-CRO).

After all PET and corresponding CT data were imported into the QDOSE software, CT images were coregistered using an automatic rigid coregistration algorithm. PET images were coupled to the CT image of the corresponding imaging time point and manually coregistered to this CT image when necessary. The frame acquisition time was adjusted from the start of the scan (standard for DICOM header) to the middle of the acquisition frame (difference of 9.6 ± 1.2 min), which appears more appropriate for pharmacokinetic evaluation.

Kidneys, liver, spleen, urinary bladder content, red marrow, heart content, and remainder of body were considered source organs. According to an established model, the red marrow activity was approximated by extrapolating activity retrieved from VOIs in lumbar vertebrae 1–5 (∼12.3% of the red marrow space) to the total red marrow (11).

Because the limbs were cropped by the limited field of view of the PET scan, the total-body cumulated activity (Ã_Total_Body_), which is important to determine the cumulated activity in the remainder of the body for dose calculations, was estimated using the injected activity (A) and the effective half-life (T_eff_) of a VOI covering most the body. Thus, the total-body cumulated activity was calculated as:

$${\text{Ã}}_{\text{Total\_Body}}=\;\left(\text{A}\cdot {\text{T}}_{\text{eff}}\right)\text{/}\left(\text{Ln}\left(2\right)\right).$$

For segmentation of the source organs, VOIs were defined for the kidneys (left and right), liver, spleen, urinary bladder, heart, lumbar vertebrae (L1–L5) and total body. Tumor areas were not considered in the segmented VOIs. Each source organ was manually segmented on the PET images at each time point, and activity values were retrieved to determine the time–activity curves for the organs. The volumes of the liver, kidneys, and spleen were determined from segmentation in the CT images. The calculation of the masses (assuming a density of 1.06 g/cm^3^) was automatically performed in QDOSE on the basis of the segmented VOIs in the CT images.

The time–activity curve for the kidneys was automatically calculated as the sum of the activities in the left and right kidneys. Monoexponential curve fitting was then applied to all organ time–activity curves. The fitted time–activity curves were then integrated from time 0 min to infinity to obtain the cumulated time–activity (Ã) values. The Ã values of the total body and red marrow were added as organs into QDOSE as external calculations for these organs were performed. The Ã of the remainder of the body was automatically calculated by subtracting the Ã of all source organs from the total-body Ã. Residence times were calculated by dividing the Ã of each source organ by the injected activity and further exported to OLINDA/EXM, version 1.1 (12), for dose calculation with this software.

Absorbed and effective dose were calculated using OLINDA/EXM (12), with the residence times exported from QDOSE. In addition, the IDAC-Dose, version 2.1, dose calculator (13) integrated in QDOSE was also used to perform dose estimations. IDAC-Dose is based on the adult reference computational phantoms of the International Commission on Radiological Protection (ICRP) (14) and on the ICRP specific absorbed fractions (15). Organ masses for the kidneys, liver, and spleen, obtained from the segmentation in the CT images, were individually adapted for each patient both in QDOSE (using IDAC-Dose) and in OLINDA/EXM to obtain more accurate dose estimations.

## RESULTS

### Adverse Events

The mean administered activity of ^18^F-FAPI-74 was 259 ± 26 MBq (range, 198–290 MBq); for the ^68^Ga-FAPI-74 examination, it was 263 MBq. After quality control, the specific activities of ^18^F-FAPI-74 were 20–50 nmol/GBq (14.7–36.8 μg/GBq); the specific activity of ^68^Ga-FAPI-74 was about 100 nmol/GBq (73.6 μg/GBq) and was only moderately worsened by physical decay during the short delay between on-site labeling and injection. Thus, the administered masses of FAPI-74 (735.8 g/mol) were about 5–40 μg per patient dose. All patients tolerated the examination well. No drug-related pharmacologic effects or physiologic responses occurred. All observed parameters remained normal and unchanged during and after the examination. No patient reported subjective symptoms during the 3.5-h observation period after tracer injection.

### Normal-Organ Biodistribution and Tumor Uptake

**FIGURE 1.**
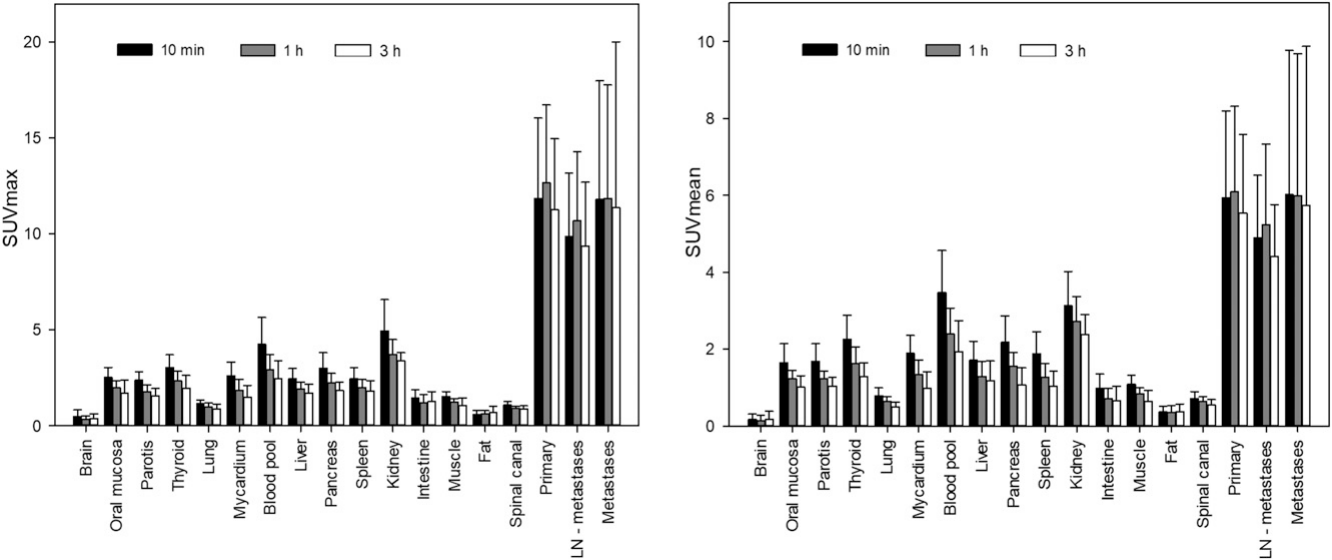
Time-dependent biodistribution of ^18^F-FAPI-74 in normal organs and tumor.

**FIGURE 2.**
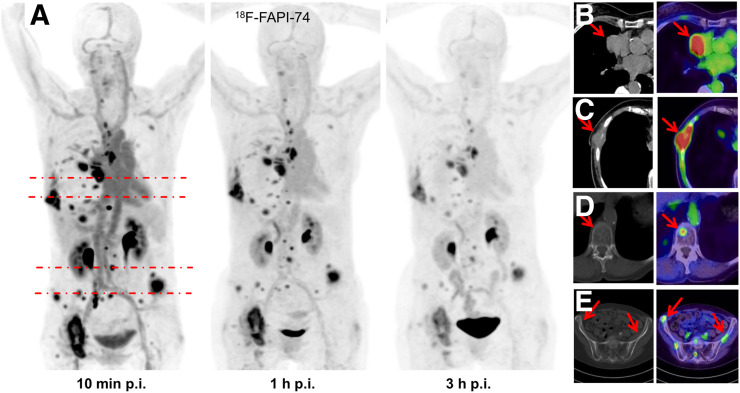
(A) Maximum-intensity projections of ^18^F-FAPI-74 PET at 10 min, 1 h, and 3 h after injection. (B) FAPI PET/CT presents favorable discrimination between tumor and myocardium. (C–E) Some FAPI-positive lesions were confirmed by CT correlate (C), whereas additional bone lesions were only detected per FAPI PET (D and E). All highlighted arrows represent FAPI uptake with morphological correlation. p.i. = after injection.

**FIGURE 3.**
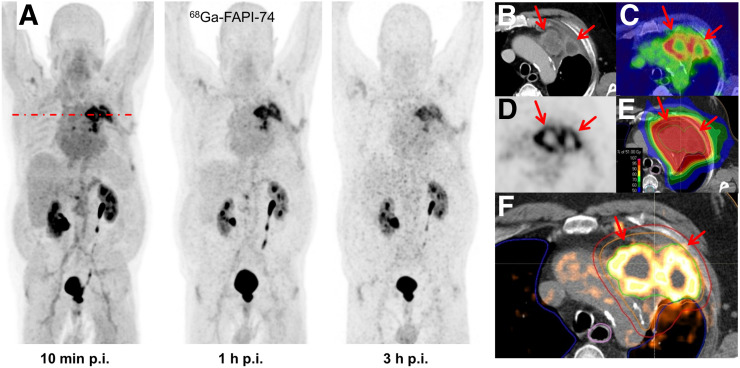
(A) Maximum-intensity projections of ^68^Ga-FAPI-74 PET at 10 min, 1 h, and 3 h after injection. (B–E) Direct comparison of contrast-enhanced CT (B), fusion imaging (C), and FAPI PET (D). (E and F) Superior tumor delineation consecutively improved dose application to tumor volume using volumetrically modulated arc therapy. Positive FAP uptake is marked by arrows (B–F). Green outline = GTV; orange outline = clinical target volume; red outline = planning target volume. p.i. = after injection.

The biodistribution of ^18^F-FAPI-74 in normal organs and tumor is presented in Figure 1 and illustrated as time-dependent maximum-intensity projections in Figure 2. In contrast to the previous ^68^Ga-FAPI-2 and ^68^Ga-FAPI-4 (5), the oral mucosa uptake did not exceed the background in muscle and connective tissue. Another difference was a moderately higher blood-pool uptake, both on the initial imaging and on the delayed imaging. Blood-pool and muscle uptake did not differ from that with ^68^Ga-FAPI-2/4, but with ^18^F-FAPI-74, vessels were definable at all time points. According to our previous FAPI tracers, there was no uptake of ^18^F-FAPI-74 in the liver or spleen exceeding the perfusion-dependent background. Within this small sample size, the tumor uptake of adenocarcinoma versus squamous cell carcinoma showed no difference, nor was it different from that found previously with ^68^Ga-FAPI-4 (16). In primary lung tumors, the average SUV_max_ was 11.8 at 10 min, 12.7 at 1 h, and 11.3 at 3 h after injection. Lymph node metastases had an SUV_max_ of 9.9 at 10 min, 10.7 at 1 h, and 9.4 at 3 h. Distant metastases demonstrated an average SUV_max_ of 11.8 at 10 min, 11.8 at 1 h, and 11.4 at 3 h. Therefore, the uptake generally peaked later than 10 min after injection, but there was already some washout from tumor tissue between 1 and 3 h after injection; therefore, the best contrast between tumor and background was achieved at 1 h after injection, and this time point was consecutively used to evaluate GTV delineation for guiding radiotherapy. The patient receiving ^68^Ga-FAPI-74 is presented in Figure 3 and presents similar kinetics, with tumor SUV_max_ being 10.4 at 10 min, 11.4 at 1 h, and 8.7 at 3 h.

### Automated Target Volume Delineation of FAPI GTVs

**FIGURE 4.**
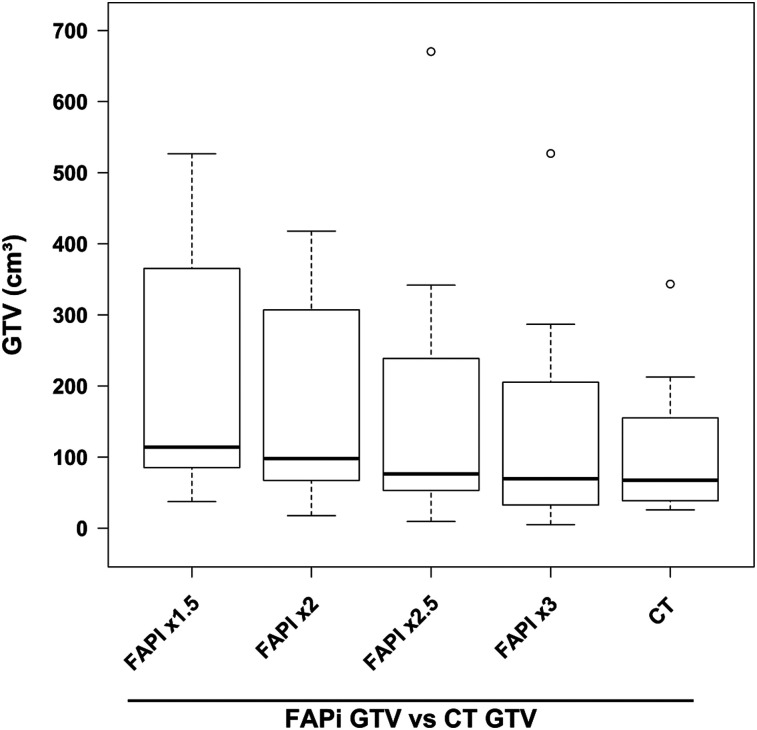
GTV automatically segmented per FAPI PET at different SUV thresholds (x = blood-pool–fold) in comparison to CT-based standard of reference.

Contouring primary lung tumors on CT resulted in a median GTV of 67.4 cm^3^ (range, 25.9–343.4 cm^3^). For a cutoff at 3-fold background, ^18^F-FAPI-74 PET traced a median GTV of 69.8 cm^3^ (*P* = 0.21; range, 5.0–527.0 cm^3^; Fig. 4). Considering a mean background SUV of 2 and a mean tumor SUV of 12, the GTVs segmented using a 3-fold background threshold are equal to GTVs segmented at 40%–50% tumor SUV_max_. In consensus with the radiation oncologist, these PET-segmented volumes were considered more likely to reflect actual tumor volumes than the corresponding CT image. One patient who was initially considered oligometastatic per the CT image was upstaged and transferred to chemotherapy after additional tumor lesions were found on ^18^F-FAPI-74 PET imaging (Fig. 2).

### Radiation Dosimetry

The OLINDA/EXM-based dosimetry estimates are presented in Table 1. Calculations according to the IDAC-Dose calculator are presented in Supplemental Table 1 (supplemental materials are available at http://jnm.snmjournals.org). For ^18^F-FAPI-74, the normalized effective dose was 1.4 ± 0.2 mSv/100 MBq (range, 1.1–1.7 mSv/100 MBq) with OLINDA/EXM and 1.2 ± 0.1 mSv/100 MBq (range, 1.0–1.4 mSv/100 MBq) with IDAC-Dose. Thus, the examinations, which were conducted with 198–290 MBq of ^18^F-FAPI-74, translated into effective doses of about 3–4 mSv per examination based on the OLINDA/EXM mean effective dose. For ^68^Ga-FAPI-74, the effective dose was 1.6 mSv/100 MBq with OLINDA/EXM and 1.4 mSv/100 MBq with IDAC-Dose. Because of a rapid renal tracer clearance and low nonspecific uptake in normal organs, the radiation dosimetry estimate of ^18^F-FAPI-74 compares favorably with most other ^18^F-labeled PET tracers in clinical use, whereas ^68^Ga-FAPI-74 is in the same range as other ^68^Ga-labeled tracers, including FAPI-2/4/46 (Table 2).

**TABLE 1:** Dose Estimates for ^18^F- and ^68^Ga-FAPI-74 According to OLINDA/EXM

Target organ	Mean ^18^F-FAPI-74 ± SD (*n* = 10)	^68^Ga-FAPI-74 (*n* = 1)
Adrenals	1.15 ± 0.09	1.29
Brain	0.78 ± 0.09	1.05
Breasts	0.78 ± 0.07	1.04
Gallbladder wall	1.17 ± 0.10	1.33
Lower large intestine wall	1.23 ± 0.16	1.31
Small intestine	1.16 ± 0.12	1.29
Stomach wall	1.06 ± 0.10	1.24
Upper large intestine wall	1.13 ± 0.11	1.27
Heart wall	2.29 ± 0.28	3.40
Kidneys	2.94 ± 0.79	3.51
Liver	1.50 ± 0.36	1.33
Lungs	0.96 ± 0.07	1.16
Muscle	0.94 ± 0.10	1.14
Ovaries	1.25 ± 0.16	1.33
Pancreas	1.18 ± 0.10	1.32
Red marrow	1.12 ± 0.11	1.11
Osteogenic cells	1.53 ± 0.14	1.70
Skin	0.73 ± 0.08	1.00
Spleen	1.67 ± 0.44	1.19
Testes	0.99 ± 0.13	1.16
Thymus	1.02 ± 0.09	1.21
Thyroid	0.91 ± 0.09	1.13
Urinary bladder wall	7.58 ± 2.84	9.86
Uterus	1.49 ± 0.25	1.46
Total body	0.97 ± 0.09	1.22
Effective dose	1.41 ± 0.22	1.61

Data are absorbed dose (mSv/100 MBq).

**TABLE 2:** Effective Dose of ^18^F-FAPI-74 and ^68^Ga-FAPI-74 in Comparison to Other PET Tracers

PET tracer	Effective dose (mSv/MBq)	Reference
^18^F-FAPI-74	0.014	This work
^68^Ga-FAPI-74	0.016	This work
^68^Ga-FAPI-2/4/46	0.008–0.015	(5,33)
^68^Ga-PSMA-11	0.023	(34)
^68^Ga-DOTATOC/-TATE	0.021	(35)
^18^F-FDG	0.020	(36)
^18^F-3′-deoxy-3′-^18^F-fluorothymidine	0.028	(37)
*O*-(2-^18^F-fluoroethyl)-l-tyrosine	0.016	(38)
(*S*)-4-(3-^18^F-fluoropropyl)-l-glutamic acid	0.032	(39)
^18^F-PSMA-1007	0.022	(40)
^18^F-flurbetaben	0.015	(41)
^18^F-flurpiridaz	0.019	(42)
^18^F-fluorocholine	0.031	(43)
^18^F-fluoromisonidazole	0.013	(44)

PSMA = prostate-specific membrane antigen.

## DISCUSSION

In this work, we evaluated the biodistribution and radiation dosimetry of ^18^F-FAPI-74 PET and demonstrated its possible value for guiding radiotherapy. In addition, labeling of ^68^Ga-FAPI-74 at ambient temperature was established, and its in vivo performance was evaluated using identical methods.

On the basis of the time-dependent biodistribution of ^68^Ga- and ^18^F-FAPI-74 in tumor and normal organs, optimal tumor-to-background ratios at limited noise were achieved by image acquisition 1 h after injection. This is in contrast to previous experience with ^68^Ga-FAPI-2/4, for which no improvement in tumor uptake between 10 min and 1 h after injection was observed. In normal organs, the time-dependent biodistribution was nearly identical to that of other quinoline-based FAPIs (5).

With a mean normalized effective dose of 1.4 mSv/100 MBq (3.5 mSv for a typical 250-MBq examination), the radiation burden of an ^18^F-FAPI-74 PET scan is lower than that of PET scans with ^18^F-FDG, the current standard in oncologic imaging (Table 2). The faint physiologic cerebral and hepatic uptake of ^18^F-FAPI-74 likely accounts for a lower radiation exposure. The effective dose of 1.6 mSv/100 MBq (3.0 mSv for a typical 185-MBq examination) for a ^68^Ga-FAPI-74 PET scan is within the range for PET imaging with ^68^Ga-FAPI-2, ^68^Ga-FAPI-4, and ^68^Ga-FAPI-46—a finding that was expected, as all share a similar biodistribution and tracer kinetics.

We used ^18^F-FAPI-74 PET/CT to plan radiotherapy in patients with lung cancer. Currently, ^18^F-FDG PET/CT is the standard for staging and target volume delineation in lung cancer. With ^18^F-FDG PET/CT, it is possible to identify additional distant metastases in about 5%–30% of patients (17), and its high sensitivity for mediastinal lymph nodes, 90%–100%, is considered sufficient to limit the target volume to involved regions. The specificity of ^18^F-FDG PET/CT is about 80% because of false-positive findings (18–24). Our preliminary experience in 11 patients is not yet sufficient to calculate the sensitivity, specificity, and accuracy of ^18^F-FAPI-74 PET/CT. However, similar to ^18^F-FDG PET, with ^18^F-FAPI-74 PET it was possible to identify additional distant metastases compared with a diagnostic CT scan (Fig. 2). In a recent case report, because of the low physiologic cerebral background uptake, ^68^Ga-FAPI-4 PET/CT made it possible to identify brain metastases from lung cancer (25). Thus, the oncologic application of ^18^F-FAPI-74 PET/CT appears promising. By applying various cutoffs, the best correlation between CT and ^18^F-FAPI-74 PET–guided GTV segmentation was found at uptakes that were 3-fold the background level, which equals 40%–50% of SUV_max_ (Fig. 4). This finding perfectly corresponds to several publications about ^18^F-FDG PET, which recommend delineating the 3-dimensional metabolic target volume at 41%–50% of SUV_max_ (26–29).

On the basis of the first DOTA-modified tracer, FAPI-2, the derivatives FAPI-4 and FAPI-46 were developed with a focus on the therapeutic option. The NOTA derivative FAPI-74 was developed as an exclusive diagnostic ligand, accepting slightly shorter tumor retention than the previous theranostic agents. Nevertheless, at early imaging time points, the diagnostic performance should be very similar. Well in line with our expectations, the tumor SUVs of ^68^Ga- and ^18^F-FAPI-74 are almost equal to that of FAPI-4 when comparing lung cancer patients (16). In a recent investigation, the accuracy of FAPI-4 PET/CT was directly compared with ^18^F-FDG PET/CT, and better tumor-to-background contrast and a higher detection rate for primary tumors, lymph nodes, and visceral metastases was found for FAPI PET than for ^18^F-FDG PET. In this study, histopathologic examination of biopsy or surgical specimens served as the gold standard for the final patient classification (30). In addition to its oncologic application, FAPI PET was also found promising for the evaluation of immune-related and heart diseases (31,32). As a practical (i.e., independent from blood sugar and physical activity), multipurpose tracer, production capacities could soon become a relevant issue. One additional advantage of FAPI-74 over previous ligands is its greater suitability for labeling with ^18^F-AlF, which would allow large-scale batch production and distribution via satellite concepts. Another characteristic of the NOTA chelator in FAPI-74 is the possibility for ^68^Ga labeling at ambient temperature. Standardized cold kits would allow chargewise constancy tests as required by regulatory bodies and would increase flexibility for local on-demand production using approved ^68^Ge/^68^Ga generators. Thus, in our center, we consider FAPI-74 to be the final evolutionary stage of diagnostic FAP-targeted ligands.

Appropriate approximation of the radiation dosimetry of a novel radiopharmaceutical is mandatory before prospective clinical trials can take place, and this investigation focused on high methodical standards for the dosimetry part, such as by considering individually segmented organ masses for all patients. Yet, only a few investigations directly comparing ^68^Ga-FAPI-2/4 versus ^18^F-FDG with histopathologic correlation have been reported (5,30). For the still-limited patient numbers that have been available so far, the accuracy of FAPI PET/CT appears promising. However, additional research evaluating the clinical impact of FAPI PET/CT for particular clinical indications, compared with a reliable standard of truth, and including sufficient patient numbers, is still pivotal.

## CONCLUSION

The high contrast and low radiation burden of ^68^Ga- and ^18^F-FAPI-74 PET/CT favor multiple clinical applications. Centralized large-scale ^18^F-AlF–based production of ^18^F-FAPI-74 or decentralized cold-kit labeling of ^68^Ga-FAPI-74 allows flexible routine use.

## DISCLOSURE

Uwe Haberkorn, Thomas Lindner, Clemens Kratochwil, and Frederik Giesel have a patent application for quinolone-based FAP-targeting agents for imaging and therapy in nuclear medicine. Uwe Haberkorn, Thomas Lindner, Clemens Kratochwil, and Frederik Giesel also have shares of a consultancy group for iTheranostics. Frederik Giesel is a medical advisor for ABX Advanced Biochemical Compound and Telix Pharmaceuticals. Sebastian Adeberg and Jürgen Debus received grants from Accuray International Sàrl, Merck Serono GmbH, and Astra Zeneca GmbH outside the submitted work. Sebastian Adeberg received grants from Novocure outside the submitted work. Jürgen Debus received grants from CRI–The Clinical Research Institute GmbH, View Ray Inc., Accuray Incorporated, RaySearch Laboratories AB, Vision RT Limited, Astellas Pharma GmbH, Solution Akademie GmbH, Ergomed PLC Surrey Research Park, Siemens Healthcare GmbH, Quintiles GmbH, Pharmaceutical Research Associates GmbH, Boehringer Ingelheim Pharma GmbH Co., PTW-Freiburg Dr. Pychlau GmbH, and Nanobiotix A.A. outside the submitted work. No other potential conflict of interest relevant to this article was reported.

**KEY POINTS**

**QUESTION:** What are the biodistribution and dosimetry characteristics of a FAPI variant that can be used for both ^18^F and ^68^Ga labeling?
**PERTINENT FINDINGS:** The NOTA chelator within the novel ligand FAPI-74 allows labeling with ^18^F-AlF, as well as the design of a cold kit for labeling with ^68^Ga. In patients with lung cancer, the new ligands presented performance and radiation dosimetry similar to previous FAPIs.
**IMPLICATIONS FOR PATIENT CARE:** FAPI-74 is our final-stage PET tracer for imaging of fibroblast-activating protein in vivo.
